# Accuracy, thoroughness, and quality of outpatient primary care documentation in the U.S. Department of Veterans Affairs

**DOI:** 10.1186/s12875-024-02501-6

**Published:** 2024-07-18

**Authors:** Michael Weiner, Mindy E. Flanagan, Katie Ernst, Ann H. Cottingham, Nicholas A. Rattray, Zamal Franks, April W. Savoy, Joy L. Lee, Richard M. Frankel

**Affiliations:** 1 Center for Health Information and Communication, Department of Veterans Affairs, Health Services Research and Development Service, Veterans Health Administration, Richard L. Roudebush VA Medical Center, CIN 13 416, Indianapolis, IN USA; 2 Department of Medicine, Indiana University School of Medicine, Indianapolis, IN USA; 3 Indiana University Center for Health Services and Outcomes Research, Regenstrief Institute, Inc, 1101 West 10th Street, 46202-4800 Indianapolis, IN USA; 4 Applied Decision Science LLC, Cincinnati, OH USA; 5 Purdue School of Engineering and Technology, Indiana University-Purdue University Indianapolis, Indianapolis, IN USA; 6 Department of Population and Quantitative Health Sciences, University of Massachusetts Chan Medical School, Worcester, MA USA

**Keywords:** Primary health care, Documentation, Electronic health records

## Abstract

**Background:**

Electronic health records (EHRs) can accelerate documentation and may enhance details of notes, or complicate documentation and introduce errors. Comprehensive assessment of documentation quality requires comparing documentation to what transpires during the clinical encounter itself. We assessed outpatient primary care notes and corresponding recorded encounters to determine accuracy, thoroughness, and several additional key measures of documentation quality.

**Methods:**

Patients and primary care clinicians across five midwestern primary care clinics of the US Department of Veterans Affairs were recruited into a prospective observational study. Clinical encounters were video-recorded and transcribed verbatim. Using the Physician Documentation Quality Instrument (PDQI-9) added to other measures, reviewers scored quality of the documentation by comparing transcripts to corresponding encounter notes. PDQI-9 items were scored from 1 to 5, with higher scores indicating higher quality.

**Results:**

Encounters (*N* = 49) among 11 clinicians were analyzed. Most issues that patients initiated in discussion were omitted from notes, and nearly half of notes referred to information or observations that could not be verified. Four notes lacked concluding assessments and plans; nine lacked information about when patients should return. Except for thoroughness, PDQI-9 items that were assessed achieved quality scores exceeding 4 of 5 points.

**Conclusions:**

Among outpatient primary care electronic records examined, most issues that patients initiated in discussion were absent from notes, and nearly half of notes referred to information or observations absent from transcripts. EHRs may contribute to certain kinds of errors. Approaches to improving documentation should consider the roles of the EHR, patient, and clinician together.

**Supplementary Information:**

The online version contains supplementary material available at 10.1186/s12875-024-02501-6.

## Background

The accuracy of clinical documentation has always been crucial for many aspects of healthcare service delivery [1]. Principles underlying good note-keeping include timeliness, completeness, usefulness, synthesis (e.g., interpretation of findings, and diagnosis), and attention to clinical plans. Clinical reasoning should be clear. The note should be readily understood by other clinicians. When documentation originates outside the encounter, its source should be identified. In the U.S., medical records determine what can be billed, and records may be referenced in a court of law. In 1995, Frankel and Beckman published on the accuracy of the medical history [2]. As noted therein, medical records are used to judge the quality of care delivered.

Electronic health records (EHRs) have transformed progress notes, from documents written *de novo* into conglomerates of checkboxes, templates, imported text, transcription from dictation, and manually generated entries. Such enhancements can speed certain aspects of work and may enhance details of notes, but may simultaneously foster errors that threaten documentation accuracy. As complexity of both EHRs and documentation requirements has grown, concern has also grown about the increasing demands that EHR time, as well as documentation policies, have placed on health professionals [3–6]. Documentation quality has implications for patient safety and reimbursement, but a comprehensive assessment of documentation quality—which includes an understanding of how events in the clinical encounter align with documentation in the medical record—requires observation or recording of the encounters, so as to know which aspects of the encounters are included in the notes, and which details in the notes are or are not verifiable in the encounters.

Few have pursued this level of detail in examining the quality of documentation in EHRs, but three reports are noteworthy. First, focusing on inpatient care, Kelly et al. reported a 19-item progress note assessment and plan evaluation tool [7], but this tool describes a note’s content based on the presence of key elements, rather than on accuracy. Second, Stetson et al. developed a nine-item Physician Documentation Quality Instrument (PDQI-9) [8]. It includes scores reflecting accuracy and thoroughness, but the authors’ own use of the instrument was limited by retrospective assessments of medical records themselves, rather than direct comparisons of documentation against the corresponding clinical encounters. Third, Weiner et al. compared documentation to encounters, by obtaining concealed audio recordings of 36 physicians interacting with one of eight trained, unannounced actors portraying one of four cases [9]. In 105 outpatient encounters, they noted 455 undocumented (incomplete or not thorough) and 181 falsely documented (inaccurate) findings. Nonetheless, they did not assess other potentially important dimensions of notes, such as presence of key elements, usefulness, report of disease status, or follow-up plans. Since these attributes may be important additional areas of focus for improving outpatient documentation, we conducted a study of clinic encounters with real patients to assess electronic documentation quality that included measures of accuracy, thoroughness, and other key elements.

## Methods

### Study design

In this prospective observational study, the quality of primary care clinicians’ electronic documentation was investigated. Notes were scored for accuracy, thoroughness, and other quality indicators, using the PDQI-9 augmented with additional measures [8, 10].

### Setting and participants

Data collection occurred in the US at four primary care clinics at a midwestern Veterans Affairs (VA) Medical Center and one associated VA community-based outpatient clinic. For many years, the VA has used its own, homegrown comprehensive EHR system, including fully computerized progress notes for clinical encounters. The VA’s system uses a single, free-form, narrative text block for its progress notes, but has optional templates that can be used with such notes, with or without customization of a template by a user of the EHR system. A template, when selected, inserts into the note its generic text (e.g., for physical examination findings) or patient-specific lists (i.e., as actually recorded in the medical record) of previously documented medical problems, current medications, allergies, vital signs, or recent laboratory test results. In this manner, a template can be used to populate an entire note, or it can be used to add to a note that is in the process of being created. Our site did not use any scribes at the time of this study; team-based writing is also not the norm in our primary care setting. Clinicians may complete progress notes during their visits or within 24 h. Ethics approval was obtained from the Indiana University Institutional Review Board prior to study recruitment.

#### Provider recruitment and participation

Both primary care providers and their patients were included. A convenience sample of 12 primary care clinicians (physicians, advanced practice nurse practitioners, or advance practice nurses) was targeted. The clinicians were approached before the start of a clinic session to obtain informed consent and collect demographic information: age, gender, years since medical or nursing school graduation, and number of years working in the institution.

#### Patient recruitment and participation

For each primary care clinician enrolled, 50 adult patient participants were targeted. English-speaking patients 18 or more years of age who had been seen in the clinic at least once before the index visit were eligible. Patients with cognitive impairment according to their provider could participate if a caregiver was present. Patients who declined participation, or who were excluded by their provider, were replaced by selecting an alternate patient. A research assistant worked with providers to verify appropriateness of study participants. Informed consent was obtained.

For each participating provider, up to five video-recorded encounters could be included in the study. Only encounters in which both the patient and clinician consented were recorded. At the discretion of the participating patient, patient-support personnel such as family, friends, or informal caregivers could be present during the encounter.

### Outcomes

#### Documentation quality measure

The PDQI-9 assesses the quality of electronic documentation on nine attributes: up-to-date, accurate, thorough, useful, organized, comprehensible, succinct, synthesized, and internally consistent. For each attribute, using a description of an ideal note, raters assigned a score from 1 (“Not at all”) to 5 (“Extremely”). For example, an *accurate* note is defined as, “The note is true. It is free of incorrect information”; a *thorough* note is defined as, “The note is complete and documents *all* [italics added] of the issues of importance to the patient.” Two attributes were excluded from our scoring procedure: first, due to the limits of our data collection that excluded other recent and pertinent notes, we did not score notes on “up-to-date”. Second, due to ambiguity in operationalizing “the note is extremely relevant” for the “useful” attribute, this attribute was excluded. The PDQI-9 has been shown to be valid and have high reliability [8]. Beyond the PDQI-9 elements themselves, the research team identified and assessed 18 additional elements thought to be important attributes or content of all notes in outpatient primary care. Presence of reason for the visit, summary of past medical history, medication list, vital signs, psychosocial concerns, assessment and plan, diagnoses, and status or severity of disease were assessed. These attributes added granularity to the PDQI-9 elements. Issues such as past medical history, medications, and diagnostic test results can be expected to be summarized in nearly all outpatient primary-care notes. In assessing accuracy and thoroughness, clinical issues were categorized as having been initiated by the patient or by the clinician. For thoroughness, issues were also categorized as biomedical or psychosocial. For example, if a patient was first to refer to food insecurity, this would be considered a patient-initiated psychosocial concern. Using six months of data prior to the interview date, timeliness of documentation was assessed by provider, by computing the percentage of notes generated during that period and completed within 24 h of the encounter. For individual notes assessed during the study period, we did not measure time to complete the note, because we could not determine how long a clinician worked on a note, and could not verify that a midstream equipment shutdown did not occur. Notes were retrieved following the 24-hour grace period for creating notes. The data collection form is provided in the Appendix.

### Video recording

For most observations, the research assistant activated the recording equipment and then left the room during the encounter; in some cases, research personnel stayed in the room to position and operate the video recording equipment. Audio recordings were used to create verbatim de-identified transcripts of the encounters.

### Data analysis

Five researchers with experience in psychology, sociology, human factors, anthropology, public health, or general internal medicine were assigned transcripts to review. Reviewers then reviewed assigned transcripts to ascertain symptoms, topics, and decisions included in the clinical encounters. All reviewers coded two encounters and discussed findings. The coding process was revised accordingly. Coding then began in pairs. The clinical note was reviewed and scored for the included PDQI-9 attributes and presence of the 18 additional elements. Accuracy and thoroughness concerns were marked against one attribute or the other, but not both. Each transcript-note set was reviewed independently by two researchers. Results were then compared in a series of discussions. All ratings were entered into Research Electronic Data Capture (REDCap), a secure, Web-based software platform [11]. Ratings for each attribute were averaged between the two raters. Ratings for the 18 elements were compared to identify disagreements. Where disagreements were identified, a third researcher reviewed the transcript-note set and served as a tie breaker. Summary statistics were calculated for attributes and elements.

## Results

Ten physicians and two nurse practitioners were recruited. One nurse practitioner was excluded due to incomplete data, so 11 providers’ data were analyzed. Recordings were collected for 49 clinical encounters. Characteristics of participants are shown in Table 1. Six providers were women. 18% of patients were African American.

**Table 1:** Characteristics of participants

Characteristic	Value
Providers (*N* = 11)	
Age, mean (years)	51
Gender, female, N (%)	6 (55)
Years since medical school, mean	22
Years in U.S. Department of Veterans Affairs, mean	13
Patients (*N* = 49)	
Gender, female, N (%)	4 (8)
Race, N (%)	
Caucasian	40 (82)
African American	8 (16)
Marital status, N (%)	
Married	31 (63)
Divorced	7 (14)
Single	7 (14)
Widowed	4 (8)
Occupational status, N (%)	
Retired	23 (47)
Employed	18 (37)
Disabled	4 (8)
Unemployed	4 (8)

Key findings are shown in Table 2. Twenty-five notes included a reason for the visit; 37 summarized the past medical history, and 32 included a medication list. Diagnostic test results were noted in 36. Assessment and plan were included in 45. Action plans were provided for all noted issues in 26 notes. For patient-initiated issues, 31 notes accurately reflected what was in the transcript; 21 did for clinician-initiated issues. Notes were judged as lacking in thoroughness—omitting one or more aspects of the encounter—in most cases except psychosocial issues initiated by the clinician. Examples of information not in the right place were past medical history combined with history of the present illness, and laboratory test results not in the “lab studies” or “data” section.

**Table 2:** Presence of documentation elements by PDQI-9 domain

Note element	Count (%) of observations (*N* = 49)
Accurate: note accurately represents information found in transcript	
For patient-initiated issues	31 (63)
For clinician-initiated issues	21 (43)
Thorough	
Note includes reason for visit	25 (51)
Note summarizes past medical history	37 (76)
Medication list is present	32 (65)
Any vital signs are noted	45 (92)
Diagnostic test results are noted	35 (74)
Transcript information from visit included in note	
Patient	
Biomedical	15 (31)
Psychosocial	22 (45)
Clinician	
Biomedical	24 (49)
Psychosocial	45 (92)
Useful	
Plan includes action targeting clinical issues at hand	
None	1 (2.0)
Some	22 (45)
All	26 (53)
Organized	
Note’s sections are named	
None	2 (4.1)
Some	12 (24)
All	35 (71)
All information in note in right place	30 (61)
Synthesized	
Note ends with assessment and plan	45 (92)
Assessment includes diagnoses or tentative diagnoses	43 (88)
Assessment includes indication of status or severity:	
None	19 (39)
Some	29 (59)
All	1 (2.0)
Note includes specific information about when patient should return	40 (82)

Overall ratings of documentation domains are summarized in Table 3. Thoroughness had the lowest mean (3.7); accuracy was second to worst (4.0). Internal consistency and comprehensibility had the best scores. Provider-based means were similar to means across all observations.

**Table 3:** Ratings of documentation, by domain

Domain	Mean (SD) rating across observations (*n* = 49)	Mean (SD) rating of provider means (*n* = 11)
Internally consistent	4.6 (0.4)	4.7 (0.2)
Comprehensible	4.5 (0.4)	4.6 (0.2)
Succinct	4.5 (0.6)	4.5 (0.4)
Organized	4.2 (0.7)	4.2 (0.5)
Synthesized	4.1 (0.6)	4.1 (0.3)
Accurate	4.0 (0.7)	4.0 (0.5)
Thorough	3.7 (0.7)	3.8 (0.5)

SD = standard deviation

**Fig. 1:**
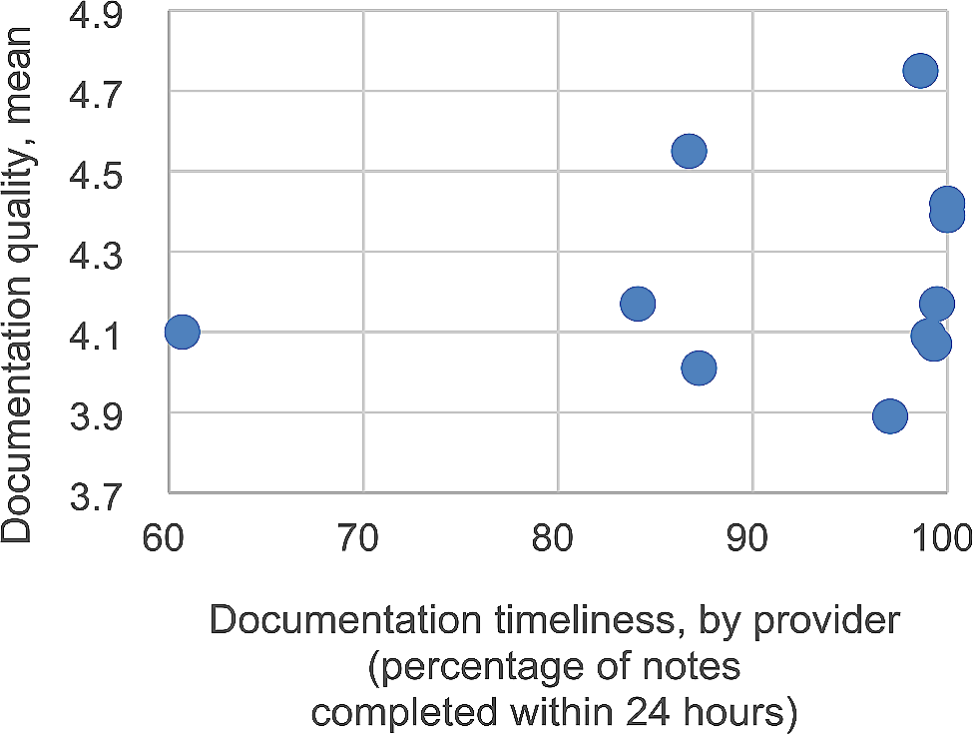
Documentation quality according to providers’ timeliness of documentation, by provider (*N* = 11). Timeliness reflects six months of data prior to the interview date

The Fig. 1 shows documentation quality according to a provider’s timeliness of documentation during a six-month period. No definitive pattern emerged upon inspection.

## Discussion

Across 49 outpatient primary care encounters examined by five reviewers, all of the assessed PDQI-9 scores, except thoroughness, exceeded 4.0, indicating good quality as well as room for improvement. Fewer than half of the issues that patients initiated were included in notes. Reasons for such omissions could include clinicians’ lack of recognition of the problem, forgetfulness while writing notes, a belief that the issue had already been addressed, or a preference to prioritize other clinical issues. Team-based writing has been described as a potential solution [12]; some have asserted that medical scribes can decrease risks to patient safety by documenting at the point of care and relieving the clinician of the burden of doing so during or after the encounter [13]. Even when clinicians themselves identify clinical issues to be addressed, delays in writing notes—often manifest as EHR use after clinic hours (though not limited to note-writing) [14]— may increase the chance of omissions. Along with total EHR time, this “outside” EHR time has increased in recent years [15].

Minimizing the time from encounter to note may help in optimizing documentation quality. Regardless of the reason for suboptimal thoroughness, the findings reinforce the potential value of techniques and verbal summaries that explicitly communicate shared understanding of key issues and their action plans before the encounter ends. The Agency for Healthcare Research and Quality developed and disseminated a “teach-back” technique for health care professionals to communicate medical information clearly and accurately to patients and families [16]. We suggest adding a reciprocal role for clinicians to *undergo* teach-back and to identify and declare *what the patient communicated to them*. Such approaches are technically simple and would quickly enable patients to identify overlooked issues that warrant attention. Including this activity would need to be weighed against other priorities; one study of U.S. office visits, for example, revealed that a median of six topics were discussed in a median visit duration of 15.6 min [17].

Perhaps of greater concern than the findings pertaining to thoroughness is the imperfect accuracy observed, with close to half of notes referring to information or observations that could not be verified by reviewing the encounter transcript. This was not strictly historical information but information that was expected to be found in transcripts themselves. A likely contributor to this problem is the use of templates [18, 19], which have become a norm in EHR systems. Some researchers have demonstrated different impacts of templates on note quality, depending on whether primary care clinicians or specialists were using them [20], or according to the specific measure being documented [21]. An additional method of speeding documentation (though without informational prompts), dictation, has been found to be comparable to, or *worse* than, other methods [21]. Some uses of templates *improve* thoroughness, probably via avoiding excessive reliance on human memory [22]. Templates can also save time by populating a note with pre-filled text, so that it does not need to be typed manually. Schnipper et al. developed a Smart Forms system to enable writing notes while capturing coded information and providing clinical decision support at the same time [23]. Although the uptake of the item form was low, it improved how issues were addressed during or after visits. Nonetheless, if all of the information represented in a template is not verified, the template can quickly result in any number of falsehoods. This may have negative consequences for billing integrity as well as for safety and effectiveness of medical care. Excessive reliance on templates might introduce biases that cause clinicians to avoid documenting specific types of issues not represented in the templates that are being used.

Psychosocial issues are common in primary care. In this study, when the clinician initiated discussion about such issues, 92% of notes included it, but when the patient initiated discussion, only 45% of notes did. This discrepancy suggests that primary care clinicians might not adequately recognize many psychosocial issues or assign enough importance to them, especially in situations where patients may provide only hints about their symptoms. Alternative explanations are that clinicians may feel unequipped to address some clinical issues related to psychosocial matters, or they may feel that other health professionals are responsible for addressing them, which could serve as a future research topic.

Structured documentation systems may lack sufficient flexibility and expressivity to address psychosocial concerns [24]. An innovative approach that could complement a teach-back strategy could be a computerized listener (audio processor) or ambient dictation technology that analyzes live conversation for “hidden” or hard-to-find issues in real time. From a more traditional perspective, the role of additional training to help clinicians identify psychosocial distress could be explored. Fanucchi and Conigliaro found that a lecture and individual feedback about progress notes did not lead to improvements in documentation quality [25]. By contrast, Habtamu and colleagues found that use of simulation and role play improved primary care clinicians’ detection of depression [26]. Other organizational, structural, or technical enhancements may be needed in designing interventions to improve note accuracy and completeness [26].

Other quality gaps raise additional questions. For example, with 8% of notes lacking an assessment and plan, were some assessments truly incomplete, and some important plans actually skipped? With 18% of notes missing follow-up plans, were some follow-up plans never arranged? With 26% lacking reports of diagnostic test results, were such results simply absent or unimportant, or were important findings unavailable, difficult to access, or overlooked? We recognize that certain variations in EHR documentation stem from authors’ preferences or styles about how to organize or structure notes [27]. At the same time, notes should not lack critical elements. Although this study did not aim to dive into additional details of these issues, further study is warranted. A longitudinal design in which recorded visits and notes could be compared with care delivery and outcomes would help answer these questions.

The study has several limitations. The sample size is small and not necessarily representative of a larger group of clinicians, whether in our institution or elsewhere. Due to the sample size, we also did not assess statistical significance of differences. The study was conducted in the VA health system, which may not be representative of other care systems. The cross-sectional design of the study did not permit us to assess the effect of continuity relationships between clinicians and patients. Some of the absences that were noted in the records of care may have been recalled by the clinician or the patient in subsequent visits. Finally, we do not have access to the clinicians’ lived experience of taking notes and whether they were aware of the differences between what was said and what was documented in the medical record. A future study would benefit from the use of cognitive task analysis or critical incident interviews with clinicians [28, 29].

## Conclusions

In summary, among outpatient primary care notes examined, fewer than half of issues that patients initiated in discussion were included in notes, and nearly half of notes referred to information or observations that could not be verified. Although EHRs have matured in certain ways, they may also contribute to a range of errors from minor to egregious. Improvements to documentation should consider the roles of the EHR, patient, and clinician together. Increasingly, documentation itself should become an active and interventional tool to improve care, instead of a passive means to archive an encounter.

### Electronic supplementary material

Below is the link to the electronic supplementary material.

Supplementary Material 1

## Data Availability

Our data are not publicly available due to the sensitivity and confidentiality of the detailed clinical notes and records that we examined.

## Abbreviations

EHR: Electronic health records
PDQI-9: Physician Documentation Quality Instrument
VA: Veterans Affairs
