# Supplementary material for: Accuracy, thoroughness, and quality of outpatient primary care documentation in the U.S. Department of Veterans Affairs
Source: BMC Prim Care. 2024 Jul 18;25:262. doi: 10.1186/s12875-024-02501-6 (PMC11264844; doi:10.1186/s12875-024-02501-6)
Supplement: Supplementary file 1 — Supplementary Material 1 [file 12875_2024_2501_MOESM1_ESM.docx]

**APPENDIX: Documentation Quality Assessment Form**

| Study ID: |  |  | Reviewer: |  |
| --- | --- | --- | --- | --- |
| Encounter date: |  |  | Review date: |  |

| For returning patients: is the patient seeing their usual (assigned) provider? | No | Yes |
| --- | --- | --- |

| **Attribute and description** | **Ratings** |
| --- | --- |
|  |  |
|  |  |
| **Accurate.** The note is true. It is free of incorrect information. | \| Actor \| **Note has extra information**  **Note** indicates something initiated by the actor that is not found in **transcript** \| \| \| --- \| --- \| --- \| \| Patient \| N \| Y \| \| Clinician \| N \| Y \|  \| Not at all accurate \|  \| Note appears to contain incorrect information \|  \| Extremely accurate:  All information in the **note** appears accurate \| \| --- \| --- \| --- \| --- \| --- \| \| 1 \| 2 \| 3 \| 4 \| 5 \| |
|  |  |

| **Thorough.** The note is complete and documents all of the issues of importance to the patient. | \| **Attribute** \| **Value** \| \| \| --- \| --- \| --- \| \| Note includes reason for visit (other than routine follow up) \| N \| Y \| \| Reason for visit appears sufficiently described in note \| N \| Y \| \| Note summarizes the past medical history (e.g., list of medical conditions) \| N \| Y \| \| Medication list is present in note \| N \| Y \| \| Any vital signs (BP, heart rate, respiratory rate, temperature) are noted in note \| N \| Y \| \| Diagnostic test results are noted in note \| N \| Y \|  \| Actor \| **Transcript has extra information**  **Transcript** indicates something actor said that is not found in **note** \| \| \| \| \| --- \| --- \| --- \| --- \| --- \| \| **Biomedical** \| \| **Psychosocial** \| \| \| Patient \| N \| Y \| N \| Y \| \| Clinician \| N \| Y \| N \| Y \|  \| Not at all thorough:  Note is missing a critical amount of information from the transcript \|  \| Note is missing some information  Transcript (+),  Note (-) \|  \| Extremely thorough:  All information in the **transcript** is also in the **note** \| \| --- \| --- \| --- \| --- \| --- \| \| 1 \| 2 \| 3 \| 4 \| 5 \| |
| --- | --- | --- | --- | --- | --- | --- | --- | --- | --- | --- | --- | --- | --- | --- | --- | --- | --- | --- | --- | --- | --- | --- | --- | --- | --- | --- | --- | --- | --- | --- | --- | --- | --- | --- | --- | --- | --- | --- | --- | --- | --- | --- | --- | --- | --- | --- | --- | --- | --- | --- | --- |
|  |  |
| **Useful.** The note is extremely relevant, providing valuable information or analysis. | \| **Attribute** \| **Value** \| \| \| \| --- \| --- \| --- \| --- \| \| **Plan** (typically at the end of note) includes **mention** of the reason for the visit \| N \| Y \| N/A \| \| **Plan** (typically at the end of note) includes **action(s)** targeting the clinical issue(s) at hand \| None \| Some \| All \| |
|  |  |

| **Organized.** The note is well-formed and structured in a way that helps the reader understand the patient’s clinical course. | \| **Attribute** \| **Value** \| \| \| \| --- \| --- \| --- \| --- \| \| **Note’s sections** **are named** (e.g., history of present illness, medications) \| None \| Some \| All \| \| All information in note appears to be in the **right place** or section (if not, note details here) \| N \| Y \|  \|  \| Not at all organized \|  \| Some sections are not named, or some information is not in the right place \|  \| Extremely organized:  All sections are named, and all information appears to be in the right place or section \| \| --- \| --- \| --- \| --- \| --- \| \| 1 \| 2 \| 3 \| 4 \| 5 \| |
| --- | --- | --- | --- | --- | --- | --- | --- | --- | --- | --- | --- | --- | --- | --- | --- | --- | --- | --- | --- | --- | --- | --- | --- |
|  |  |

| **Comprehensible.** The note is clear, without ambiguity or sections that are difficult to understand. | \| Not at all comprehensible \|  \| Some ambiguities, or difficulty in understanding the note \|  \| Extremely comprehensible \| \| --- \| --- \| --- \| --- \| --- \| \| 1 \| 2 \| 3 \| 4 \| 5 \| |
| --- | --- | --- | --- | --- | --- | --- | --- | --- | --- | --- | --- |
|  |  |

| **Succinct.** The note is brief, to the point, and without redundancy. | \| Not at all succinct \|  \|  \|  \| Extremely succinct:  Entirely appropriate in length (neither too short nor too long) \| \| --- \| --- \| --- \| --- \| --- \| \| 1 \| 2 \| 3 \| 4 \| 5 \| |
| --- | --- | --- | --- | --- | --- | --- | --- | --- | --- | --- | --- |
|  |  |
| **Synthesized.** The note reflects the author’s understanding of the patient’s status, and ability to develop a plan of care. | \| **Attribute** \| **Value** \| \| \| \| --- \| --- \| --- \| --- \| \| Note ends with assessment and plan (aside from automated text based on reminders) \| N \| Y \|  \| \| Assessment includes diagnoses or tentative or possible diagnoses (e.g., a differential diagnosis) \| N \| Y \|  \| \| Assessment includes indication of status or severity of disease (e.g., uncontrolled, mild, severe) \| None \| Some \| All \| \| Note includes specific information about when the patient should return or seek follow-up care (e.g., return in 6 months) \| N \| Y \|  \|  \| Not at all synthesized \|  \|  \|  \| Extremely synthesized:  Author has summarized understanding of patient’s status, and provided a corresponding plan of care \| \| --- \| --- \| --- \| --- \| --- \| \| 1 \| 2 \| 3 \| 4 \| 5 \| |
|  |  |

| **Internally consistent.** No part of the note ignores or contradicts any other part. | \| Not at all internally consistent \|  \| Some contradictions \|  \| Extremely internally consistent:  No contradictions \| \| --- \| --- \| --- \| --- \| --- \| \| 1 \| 2 \| 3 \| 4 \| 5 \| |
| --- | --- | --- | --- | --- | --- | --- | --- | --- | --- | --- | --- |
| **Comments (optional)** |  |
